# Development of *Leishmania* vaccines: predicting the future from past and present experience

**DOI:** 10.7555/JBR.27.20120064

**Published:** 2012-09-30

**Authors:** Joshua Muli Mutiso, John Chege Macharia, Maria Ndunge Kiio, James Maina Ichagichu, Hitler Rikoi, Michael Muita Gicheru

**Affiliations:** a Department of Tropical and Infectious Diseases, Institute of Primate Research, Karen, Nairobi 24481-00502, Kenya;; b Department of Zoological Sciences, Kenyatta University, Nairobi 43844-00100, Kenya.

**Keywords:** *Leishmania*, leishmaniasis, vaccine, immunization, immune response

## Abstract

Leishmaniasis is a disease that ranges in severity from skin lesions to serious disfigurement and fatal systemic infection. Resistance to infection is associated with a T-helper-1 immune response that activates macrophages to kill the intracellular parasite in a nitric oxide-dependent manner. Conversely, disease progression is generally associated with a T-helper-2 response that activates humoral immunity. Current control is based on chemotherapeutic treatments which are expensive, toxic and associated with high relapse and resistance rates. Vaccination remains the best hope for control of all forms of the disease, and the development of a safe, effective and affordable antileishmanial vaccine is a critical global public-health priority. Extensive evidence from studies in animal models indicates that solid protection can be achieved by immunization with defined subunit vaccines or live-attenuated strains of *Leishmania*. However, to date, no vaccine is available despite substantial efforts by many laboratories. Major impediments in *Leishmania* vaccine development include: lack of adequate funding from national and international agencies, problems related to the translation of data from animal models to human disease, and the transition from the laboratory to the field. Furthermore, a thorough understanding of protective immune responses and generation and maintenance of the immunological memory, an important but least-studied aspect of antiparasitic vaccine development, during *Leishmania* infection is needed. This review focuses on the progress of the search for an effective vaccine against human and canine leishmaniasis.

## INTRODUCTION

*Leishmania* parasites cause a wide variety of diseases that range in severity from self-healing cutaneous leishmaniasis (CL) to fatal disseminated visceral leishmaniasis (VL)[1]. The disease manifestation is determined by both the species of *Leishmania* and the host immune system, although certain species are associated with specific clinical conditions. For example, VL usually results from infection with either *Leishmania donovani* (*L. donovani*) or *Leishmania infantum* (*L. infantum*)[2]. The leishmaniases are responsible for the second-highest number of deaths due to parasitic infection globally[3]. They have an overwhelming estimated prevalence of 15 million infected humans, and cause a burden estimated at 2,357,000 disability-adjusted life years. Leishmaniasis has been classified as one of the most neglected diseases; the estimated disease burden places it second in mortality and fourth in morbidity among the tropical infections[4]. The disease is prevalent in Africa, Latin America, Asia, the Mediterranean basin and the Middle East, and recently has been identified in East Timor[5] and Thailand[6]. Diagnosis is based on clinical criteria used for humans: lesion histopathology, detection and isolation of parasites from the lesions, and the analysis of the small subunit ribosomal RNA genes using the polymerase chain reaction (PCR)[7]. Although chemotherapeutic treatments for the leishmaniases exist, the drugs are costly, limited and toxic[8]. Furthermore, available treatments are threatened by drug resistance, which is reviewed by Croft et al.[9]. Even with treatment, various disease forms can cause lifelong disfigurement and scarring. Thus, as with all infectious diseases, prevention of leishmaniasis is superior to a cure. A prophylactic vaccination would prove to be the most effective strategy to control infection and spread of this group of diseases[10]. However, despite substantial effort spent in developing a vaccine, there is currently no licensed vaccine against human leishmaniasis[11]. One complication in the control of human leishmaniasis is the existence of *Leishmania* animal reservoirs, especially the domestic dog, which is associated with widespread CVL in South America[12] and the Mediterranean region[13]. The development of an effective CVL vaccine may help in controlling zoonotic VL infection in humans.

Host resistance to *Leishmania* infection is mediated by cellular immune responses leading to macrophage activation and parasite killing. Antileishmanial immunity is mediated by both innate and adaptive immune responses and requires effective activation of macrophages, dendritic cells (DCs), and antigen-specific CD4^+^ and CD8^+^ T cells[14]. Acquired immunity in murine CL caused by *Leishmania major* (*L. major)* is mediated by parasite-induced production of IF-γ by CD4^+^ T cells (Th1 subset), and can develop in the absence of CD8^+^ T cells[15]. However, both CD4^+^ and CD8^+^ T cells are required for an effective defense against murine visceral *L. donovani* infection[16]–[18]. Antileishmanial antibodies, which are produced at low level in CL and at a very high level in VL, play no role in protection. A high antibody level is a marker of progressive disease in VL[18]. Effector CD4^+^ T cells are responsible for the production of cytokines critical for the activation of macrophages and are required for optimal host response to infection[17]. Cytotoxic CD8^+^ T cells also play a host protective role and are required for the effective clearance of parasites[16] and the generation of memory responses[19]. Interestingly, 80%-90% of human infections are subclinical or asymptomatic, and this asymptomatic infection is associated with strong cell-mediated immunity. Only a small percentage of infected individuals develop severe disease, and patients who recover from leishmaniasis display resistance to reinfection[18]. This suggests the development of clinical immunity and provides a biological rationale for the development of vaccines that impart a strong cellular immunity.

Amongst the various types of leishmaniases, VL is the most dreaded and devastating[20]. Humans are the only known hosts for *L. donovani*; however, a complication in the control of leishmaniasis is the involvement of animal reservoirs. *L. infantum* is primarily a zoonotic disease and canine species are the main animal reservoirs. CVL affects millions of dogs in Europe, Asia, North Africa, and South America and has been associated with outbreaks of human VL[12]. Both symptomatic and asymptomatic *Leishmania*-infected dogs act as a source of parasites for VL transmission[21], and CVL represents a significant public health issue. A current approach to breaking the VL transmission cycle is the development of CVL vaccine, which may be crucial for controlling VL infection in human populations. Experimental infection models are used to screen and evaluate *Leishmania* vaccines, and several animal species have been used including mice, hamsters, monkeys, and dogs[22]. However, no single in vivo model accurately reflects all aspects of human VL disease, which has been a major limitation in the development of VL vaccines. However, a vaccine for man requires a final evaluation in a non-human primate model before clinical application. This is due to the closer phylogenetic relationship between man and NHP model, as compared to other animal models. The precise immune mechanisms underlying human VL are still not fully understood, and the responses necessary for protection by vaccination in experimental infection models may not reflect those required for efficacy in endemic areas[23]. Improved understanding of these areas should assist in the development of an effective prophylactic vaccine, capable of inducing the specific immune responses required to battle this parasite. The profile of an antileishmanial vaccine would need to incorporate several important features, such as safety, ease of production at a low cost in endemic countries, the induction of robust, long-term T cell responses, and both prophylactic and therapeutic efficacy.

## DEVELOPMENT OF *LEISHMANIA* VACCINES

Evidence that most individuals who were once infected with *Leishmania* are resistant to clinical infections when later exposed provides the justification for vaccine development[11]. Although there is no licensed vaccine against any form of leishmaniasis for general human use, a vaccine against different forms should, in theory, be possible. This is considering the abundance of genetic and biological information about the parasite, clinical and experimental immunology of the disease, and the availability of vaccines that offer protection in experimental animals against challenge with different *Leishmania* species[24]. The leishmaniases are unique among parasitic diseases because a single vaccine could successfully prevent and treat disease and has the potential to protect against more than one *Leishmania* parasite species[25]. To date, there have been numerous attempts at developing a successful vaccine against leishmaniasis and there are several categories of vaccine candidates. Development of vaccines can be divided into five different stages: discovery, pre-clinical development, clinical development, registration, and postmarketing evaluation. For leishmaniasis vaccines, there has been much activity in the discovery area yielding many candidate second generation vaccines. In general, the leishmaniasis vaccines in development can be divided into various categories: (i) Live *Leishmania* vaccine (*Leishmanization*, LZ); (ii) First generation vaccines consisting of whole killed *Leishmania* or fractions of the parasite; (iii) Second generation vaccines including all defined vaccines, i.e., recombinant proteins, DNA vaccines and combinations thereof; (iv) Live-attenuated *Leishmania* vaccines. A review on LZ, first generation vaccines, selected “most important” recombinant protein vaccine candidates, DNA and live-attenuated *Leishmania* vaccines is presented.

### Live *Leishmania* vaccine (Leishmanization, LZ)

From ancient times, it was known in western and south-western Asia that recovery from CL is followed by a strong immunity to the disease[26]. Hence, much like cow-pox, the exudates from active lesions were inoculated into a covered part of the body of healthy children to induce a self-healing lesion and protection against multiple lesions on the face and other exposed parts of the body. This approach later became known as ‘leishmanization’ (LZ) and live virulent *L. major* promastigotes were harvested from free cultures and used in large-scale vaccination trials during the 1970s and 1980s in Israel, Iran and the former Soviet Union[24],[27]–[29]. Although still practiced in Uzbekistan, the observation of adverse side effects, including the development of large persistent lesions, psoriasis and immunosuppression, led to the discontinuation of LZ in many countries. The focus of vaccine development consequently shifted towards killed organisms[25],[26]. Nowadays, the development of a new vaccine must meet several strict criteria where safety, reproducibility and efficacy are of utmost importance[24]. Although LZ gave a high percentage of successful lesion development and subsequent immunity to *L. major* infection, it was neither reproducible nor safe. Furthermore, the viability and infectivity of the injected parasites varied and organisms without virulence induced delayed-type hypersensitivity (DTH)[29]. Another approach to live non-attenuated vaccines was taken by Breton et al.[30] who used a non-pathogenic species, *Leishmania tarentolae* (*L. tarentolae*), to immunize mice. The high level of immunological cross-reactivity between species at both the humoral and cellular level provides the rationale for using non-pathogenic *Leishmania* species to immunize against virulent species[31]. Breton et al.[30] has indicated that this approach may be promising as a vaccine against murine *L. donovani* VL. Vaccinated BALB/c mice were able to yield a protective immune response after only a single intraperitoneal vaccination. Although the results from this study appear promising, vaccination with live non-attenuated parasites is not an appealing prospect. At present, there is only one prophylactic live vaccine in use. This is a mixture of live virulent *L. major* mixed with killed parasite registered in Uzbekistan. The parasite is isolated from an active lesion to produce the vaccine each year to overcome the problem of loss of virulence and given a few months prior to the start of transmission cycle to high risk population[32],[33]. The problems associated with live vaccines are standardization and quality control. There may also be concerns about the risk of HIV transimission and hence the attenuation of live parasites to render them avirulent may offer a safer, more stable approach to live vaccination.

### First generation vaccines (Whole killed or fractions of the *Leishmania* parasite)

#### Whole killed parasites - New world

The early trials with killed *Leishmania* as a vaccine were conducted in Brazil in the 1940s. Later, from the 1970s onwards Mayrink and his colleagues developed a killed vaccine composed of five isolates of *Leishmania* containing four different species[34]. This was simplified to a single (*Leishmania amazonensis*, *L. amazonensis*) vaccine and tested for prophylactic potential in Columbia[35],[36] and Ecuador[37] and, as an adjunct to chemotherapy in Brazil. Convit and his group in Venezuela introduced their autoclaved *Leishmania mexicana* (*L. mexicana*) + BCG for immunotherapy and/or immunochemotherapy[38]. Several prophylactic studies were done[34] with inconclusive results or low protection induced by the vaccine (killed *Leishmania* injected 3 times without any adjuvant) when given to leishmanin-negative (Montenegro skin test, MST the same as leishmanin skin test, LST) individuals. However, a highly significant finding of this group, which has been confirmed repeatedly by others, is that the incidence rate amongst the MST converted individuals in the vaccine group was significantly lower than those in the control (unvaccinated) group or vaccinated but MST nonconverted individuals. As in Venezuela with Convit's vaccine, Mayrink's vaccine was effective in reducing the dose of antimony required to achieve cure[39]. Based on these trials, the vaccine was registered as an adjunct to antimony therapy in Brazil, but not for prophylactic use.

In Venezuela, autoclaved killed *L. mexicana* is now used to treat patients with CL. If the patient does not respond after three injections (2 months), antimony treatment is initiated[40]. In Ecuador, two doses of a vaccine composed of *L. amazonensis* and *L. mexicana* mixed with BCG induced 73% protection[41]. A double-blind randomized efficacy trial with Mayrink's vaccine, formulated to be injected intradermally mixed with BCG, could not be conducted as planned due to flooding caused by E1 Niño. As a result, case finding was delayed and it was not possible to determine the efficacy of the vaccine, if any[37]. This study, unlike the claims of the authors, should be considered as “inconclusive”. A comparative trial of this vaccine, with and without BCG, was conducted in Colombia. The formulation without BCG was chosen to proceed to efficacy trial due to lesion formation at the site of injection with BCG[35]. A randomized double-blind placebo control efficacy trial against natural infection was conducted in Colombia[36]. Similar to the finding of Mayrink[34] in Brazil, there was no significant difference in incidence rates of vaccinated vs unvaccinated controls. Unlike Mayrink's studies, there was no skin test performed after vaccination, hence the immunogenicity of the vaccine soon after immunization was not known. A low conversion rate, due to whatever reason, could account for lack of sufficient efficacy. Furthermore, it was not possible to determine the incidence only in those vaccinated individuals who responded by skin test conversion, the group that in previous trials were shown to have a lower incidence rate. In general considering all trials, based on the immunogenicity of various killed *Leishmania* preparations, it seems that a better adjuvant than BCG would be required to produce a potent vaccine.

#### Whole killed parasites - Old world

Zoonotic, as well as anthroponotic CL exist at very high incidence rates in different parts of Iran, which makes the disease one of the major health problems in this country. This has given the impetus to focus much attention and resources for developing a vaccine during the last decade. Using the same organism that was used for LZ in Iran, a seed bank and several hundred seed lots were prepared at Razi Serum and Vaccine Research Institute, Hesarak, Iran. Several procedures were used for killing the promastigotes and finally autoclaving was selected, similar to that developed in Venezuela to produce autoclaved *L. major* (ALM) as the vaccine. Stepwise phase I-II safety and dose-response trials were conducted in non-endemic area[42],[43]. A dose of 1.0 mg ALM mixed with one tenth of the dose of BCG usually used for antituberculosis vaccine was chosen for further development. The results showed that the mixture was safe, acceptable and induced LST conversion in about 38% and weak but measurable IFN-γ production. Efficacy trials of a single dose of 1 mg of ALM mixed with one tenth dose of BCG were conducted in a zoonotic[44] as well as anthroponotic foci of Iran[45]. The immunogenicity of the vaccine in the field was much reduced and only 16.5% LST conversion was seen in the anthroponotic focus of Bam, Iran. To increase immunogenicity, multiple injections were planned. Safety and immunogenicity of multiple doses were studied in non-endemic foci before proceeding to field efficacy trials. Two doses of the vaccine reduced the incidence by 43% in LST converted volunteers in Sudan against VL involving 2306 volunteers[46]. In earlier studies, Indian langur monkeys were vaccinated with autoclaved *L. major* plus BCG against *L. donovani*[47]. A triple dose schedule each of 1 mg ALM + 1 mg BCG was more efective than a single dose vaccination. The triple dose vaccinated animals showed significantly reduced parasite numbers as compared to the single dose vaccinated animals. It was decided that the vaccine was not immunogenic enough and another adjuvant was sought.

To enhance the immunogenicity of the ALM+BCG vaccine, ALM was adsorbed to alum (aluminum hydroxide), and the resulting alum-ALM was mixed with BCG just prior to injection. Indeed, addition of alum to ALM led to enhanced immunogenicity as a single injection of killed *Leishmania* in alum plus IL-12 induced strong cellular immune responses and protection in Rhesus monkeys against CL[48]. Further vaccination studies utilizing the vervet monkey model used the ALM in conjunction with IL-12 as an adjuvant and the results indicated type 1 immune responses which failed to protect vaccinated animals against *L. Major* challenge[49]. However, alum-ALM + BCG protected Langur monkeys against VL[50]. A single injection of alum-ALM mixed with BCG showed 70% protection in canine leishmaniasis in Iran. The vaccine did not produce high titres of antileishmanial antibodies and the efficacy was assessed by raising serum antibodies after exposure to natural infection[51]. A dose escalation trial of a single intradermal injection of alum-ALM (10, 100, 200 or 400 µg of *Leishmania* proteins) mixed with BCG was carried out in healthy volunteers from a non-endemic area of Sudan. The results showed the highest skin test conversion seen in any *Leishmania* vaccine trials so far[52]. With the exception of the 200 µg arm, all volunteers developed a strong LST reaction, which remained strongly positive up to 90 days post vaccination. Local side effects were minimal and acceptable and no systemic side effect was recorded. The low immune response in the 200 µg group was attributed to the problems with the BCG vial used on that day as BCG lesions either did not develop or developed many weeks after injection. The safety-immunogenicity trial was repeated in Sudan and essentially all participants responded strongly with a single injection[53]. Recently, whole killed formalin-fixed *L. major* promastigotes (KLM) were delivered in the BALB/c model of CL. The vaccine was comprised of KLM in conjunction with alum, BCG or montanide ISA 720 (MISA) as adjuvants and there was significant protective type 1 immune response in mice immunized with BCG+KLM or MISA+KLM[54]. However, the study indicated adverse reactions following immunization with BCG vaccine. More recently, these murine data were evaluated in the vervet monkey model of visceral *L. donovani* leishmaniasis, where animals were vaccinated with *L. donovani* whole sonicate antigen with or without alum-BCG, monophosphoryl lipid A or MISA 720[55]. The results indicated strong type 1 immune responses in animal groups immunized with either alum-BCG+Ag or MISA+Ag, with the MISA+Ag vaccinated animals having better results than the alum-BCG+Ag group. However, vaccination with alum-BCG was associated with adverse local reactions, which may negatively influence the application of BCG as an adjuvant in *Leishmania* vaccines. Currently, an immunochemotherapy trial of 4 injections of 100 µg of alum-ALM+BCG combined with sodium stibogluconate (Pentostam) for treatment of post-kala-azar dermal leishmaniasis (PKDL) is underway in Sudan. The initial results are highly encouraging with significant cure in the group receiving combined therapy compared to chemotherapy alone[56]. Thus, alum precipitated ALM mixed with one tenth dose of BCG appears to constitute a safe vaccine and an appropriate candidate for further development.

#### Fractionated Leishmania vaccine preparation

The fucose mannose ligand (FML) antigen is present on the surface of the parasite throughout the life cycle, and it has been shown that it is a potent immunogen in mice and rabbits and a sensitive, predictive and specific antigen in serodiagnosis of human and canine kalaazar[57]. The FML saponin formulation is shown to be safe, immunogenic and protective in BALB/c, Swiss albino mice and CB hamsters[58]. In a Brazillian region endemic for both human and dog VL, efficacy trials using the FML vaccine in dogs induced 92 and 95 percent protection in naturally exposed vaccinated dogs. Protection induced by the FML-*Quillaja* saponin vaccine lasted up to 3.5 yr after vaccination; therefore, it induced strong protective effect against canine kala-azar in the field[59]. Soluble *L. donovani* antigens (Lag) in conjunction with positively charged liposomes was evaluated as a candidate vaccine against murine and hamster-*L. donovani* challenge[60]. Intraperitoneal immunizations of hamsters and BALB/c mice with the leishmanial antigens confered protection against infection with the virulent promastigotes. These protected animals elicited profound DTH and increased levels of *Leishmania*-specific IgG antibodies. Robust levels of induced IgG2a antibodies following immunization with liposome-encapsulated antigens seemed to be responsible for the significant levels of resistance against the disease. In another study, *L. major* culture-derived soluble exogenous antigens (SEAgs) delivered alone or in conjunction with alum, recombinant murine interleukin-12 (rmIL-12), alum and rmIL-12 or montanide ISA 720 (MISA 720) were used as a vaccine against *L. major* challenge in BALB/c mice[61]. Data indicated that mice immunized with the SEAgs alone showed significant T cell proliferation and secreted a mixed profile of type 1 and 2 cytokines. This immune response was associated with significant reduction in lesion sizes with more than 100-fold fewer parasites as compared to other groups combining SEAgs with adjuvants. Western blot analyses of SEAgs revealed the presence of lipophosphoglycan and gp46/M2/PS-2 in the secreted antigens. In a more recent study, a vaccine comprising of *L. donovani* promastigote soluble antigens (sLAg) encapsulated in non-phosphatidylcholine (non-PC) liposomes prepared from *Escherichia coli (E. coli)* lipids was evaluated in BALB/c mice and hamsters[62]. The non-PC liposome entrapped promastigote antigens elicited parasite specific CD8^+^ and CD4^+^ T-cell immune response and protected immunized animals against VL. The vaccine administration induced strong humoral, as well as cell mediated immune responses in immunized animals. Recently, purified soluble *L. major* antigen (SLA) was used as a model of first generation vaccine with the nuclease-resistant phosphorothioate CpG oligodeoxynucleotides (PS CpG) or nuclease-sensitive phosphodiester CpG ODNs (PO CpG) as adjuvants in murine CL[63]. This vaccine, used in liposomal delivery system, showed a high protection rate compared with the control groups with no significant difference in immune response generation between mice immunized with PS CpG and the group receiving PO CpG.

### Second generation vaccines (recombinant protein and DNA vaccines)

#### Recombinant protein vaccines

The newer vaccines under consideration comprise recombinant DNA-derived antigens and peptides. Some of the target antigens are species and life cycle stage specific, while others are shared by promastigotes and amastigotes. Some are conserved among *Leishmania* species, while others are not. Since T cells recognize peptides derived from cytosolic proteins bound in the MHC class I groove or peptides derived from the lysosomal compartment bound in the MHC class II groove on the antigen-presenting cell surface, it would appear that virtually any parasite protein might function as an antigen regardless of its location in the parasite. At the effector stage in the lesion, it may not be important if the antigens are present on the surface of infected or bystander antigen-presenting cells. As long as the appropriate proinflammatory Th1 cytokines are generated in the lesion, macrophage activation and parasite killing should occur. A variety of *Leishmania* vaccines consist of recombinant proteins; poly-proteins produced by DNA cloning. More recent efforts aim at increasing the immunogenicity of DNA cloning vaccines, including the use of genetic adjuvants and plasmid-based expression of viral replicons[64]. Some of the important recombinant protein candidate vaccines include: surface expressed glycoprotein leishmaniolysin (gp63), *Leishmania* activated C kinase (LACK), parasite surface antigen (PSA), *Leishmania*-derived recombinant polyprotein (*Leish*-111f) and serine proteases among other candidate antigens[65].

Native and recombinant forms of *Leishmania* gp63 have been used as candidate vaccine against both CL and VL. Anti-gp63 antibodies and DTH reaction were recorded in vervet monkeys vaccinated with a triple dose totalling 150 µg of recombinant gp63 mixed with BCG as an adjuvant, resulting in partial protection of immunized animals[66]. Although gp63 produced in *E. coli* was safe, peripheral blood leucocytes (PBL) from these animals neither proliferated nor produced any IFN-γ following in vitro stimulation with the antigen. In a different study aimed at evaluating the role of CpG ODN co-encapsulated with rgp63 antigen in cationic liposomes (Lip-rgp63-CpG ODN) in immune response enhancement and protection in BALB/c mice against leishmaniasis, Lip-rgp63-CpG ODN prepared by using the dehydration-rehydration vesicle (DRV) method significantly inhibited *Leishmania major* infection in mice, measured by footpad swelling, compared to Lip-rgp63, rgp63 alone, rgp63 plus CpG ODN, PBS or control liposomes[67]. Mice immunized with Lip-rgp63-CpG ODN also showed the lowest spleen parasite burden, the highest IgG2a/IgG1 ratio and IFN-γ production and the lowest IL-4 production compared to the other groups. The results indicated that co-encapsulation of CpG ODN in liposomes improves the immunogenicity of *Leishmania* antigen.

In a different study, human T lymphocyte responses to *L. amazonensis* native and recombinant gp63 (rgp63) produced in *E. coli* were evaluated in individuals with active or cured CL, mucosal *Leishmaniasis* or VL. T cell lines generated against rgp63 proliferated, but failed to proliferate in response to native gp63 or to promastigote lysate. Thus, rgp63 was effective in eliciting T cell responses from patients with active or cured *Leishmania* infection[68]. In a recent study, gp63 in stable cationic distearoyl phosphatidylcholine (DSPC) liposomes conferred sustained vaccine immunity to susceptible BALB/c mice infected with *L. donovani*[69]. Production of IFN-γ and IL-4 by splenic T cells, and of serum immunoglobulin G1 (IgG1) and IgG2a following immunization, suggested that the vaccine induced a mixed Th1/Th2 response. However, control of disease progression and parasitic burden in mice vaccinated with gp63 in the cationic DSPC liposomes was associated with enhancement of antigen-specific IFN-γ and downregulation of IL-4, demonstrating a Th1 bias. In a different study, gp63 as a recombinant antigen combined with liposome formulations with different bilayer compositions consisting of egg phosphatidylcholine (EPC, Tc < 0°C), dipalmitoylphosphatidylcholine (DPPC, Tc 41°C), or distearoylphosphatidylcholine (DSPC, Tc 54°C) was used to immunize mice against cutaneous *L. major* infection[70]. The results indicated that the generated immune response in mice was influenced by the bilayer composition of liposomes, so that mice immunized with liposomes consisting of EPC induced a Th2 type of immune response while liposome consisting of DPPC or DSPC induced Th1 type of immune response. Working on DNA based vaccines against *L. donovani*, Mazumder et al.[71] compared the potency, efficacy, and durability of DNA, protein and heterologous prime-boost (HPB) preparation, and major surface gp63 cloned into mammalian expression vector pcDNA3.1. The researchers demonstrated that gp63 DNA based vaccination induced immune responses and conferred protection against challenge infection, while HPB based on gp63 in association with the vaccine adjuvant Cpg ODN emphasized cellular and humoral responses that correlated with durable protection against a challenge with *L. donovani* up to 12 weeks post vaccination.

*Leishmania* parasite surface antigen-2 (PSA-2) is a family of glycoinositol phospholipids anchored glycoprotein (GPI-gp46) expressed in both promastigote and amastigote of all *Leishmania* species except *Leishmania braziliensis* (*L. braziliensis*)[72]. Parasite surface antigen-2 is comprised of three polypeptides with approximate molecular weights of 96,000, 80,000 and 50,000 kDa[73]. The amastigote form expresses a distinct but closely related PSA-2 with a molecular weight of 50,000 kDa. It has been shown that vaccination with native PSA-2 with *Corynebacterium parvum* as adjuvant protects mice from *Leishmania* through a Th1 mediated response, but the recombinant PSA-2 purified from *E. coli* and administered in immuno-stimulating complexes (ISCOMs) or mixed with *C. parvum* as an adjuvant does not induce protective immunity despite the induction of Th1 responses. Both C3H and BALB/c mice showed good protection against *L. major* challenge when the DNA was administered as a prophylactic vaccine, although significant healing from established *L. major* infection was seen when the plasmid was given as an immunotherapeutic agent[74]. Parasite surface antigen-2 is involved in macrophage invasion through the interaction of its leucine rich repeats (LRR) with complement receptor 3 (CR3). In a study that was the first to demonstrate the functional role for PSA-2, the authors indicated that in addition to leishmanolysin and LPG, parasite attachment and invasion of macrophages involve a third ligand comprising the LRRs shared by PSA-2 and PPG and that these interactions occur via the CR3[75].

Mucin-like glycoproteins called proteophosphoglycans (PPGs) exist as secretory as well as surface-bound forms in both promastigotes and amastigotes. The structure and function of PPGs have been reported to be species and stage specific as in the case of *L. major* and *L. mexicana* but not for *L. donovani*. The DNA-encoding N-terminal domain of the *PPG* gene was evaluated as a vaccine in golden hamsters (*Mesocricetus auratus*) against *L. donovani* challenge. A prophylactic efficacy of approximately 80% was observed in vaccinated hamsters and all of them could survive beyond six months after challenge. The efficacy was supported by a surge in inducible nitric oxide synthase (iNOS), IFN-γ, TNF-α, and IL-12 mRNA levels along with extreme downregulation of transforming growth factor beta (TGF-β), IL-4, and IL-10. A rise in the level of *Leishmania*-specific IgG2 was also observed, which was indicative of enhanced cellular immune response. The results suggested the N-terminal domain of *L. donovani* PPG as a potential DNA vaccine against VL[76]. In an attempt to select candidate antigens for a vaccine protecting against different *Leishmania* species, the efficacy of vaccination using *Leishmania* ribosomal proteins and saponin as adjuvant was examined in BALB/c mice against challenge infection with both parasite species[77]. Mice vaccinated with parasite ribosomal proteins purified from *L. infantum* plus saponin showed a specific production of IFN-γ, IL-12 and GM-CSF after in vitro stimulation with *L. infantum* ribosomal proteins. Vaccinated mice showed a reduction in the liver and spleen parasite burdens after *Leishmania chagasi* (*L. chagasi*) infection. After *L. amazonensis* challenge, vaccinated mice showed a decrease of dermal pathology and a reduction in the parasite loads in the footpad and spleen. In both models, protection was correlated with an IL-12-dependent production of IFN-γ by CD4^+^ and CD8^+^ T cells that activate macrophages for the synthesis of NO. In the protected mice, a decrease in the parasite-mediated IL-4 and IL-10 responses was also observed. In mice challenged with *L. amazonensis*, lower levels of anti-parasite-specific antibodies were detected. The study concluded that *Leishmania* ribosomal proteins plus saponin fits the requirements to construct a pan-*Leishmania* vaccine[77]. A different study has demonstrated that vaccination with ribosomal protein extracts administered in combination with CpG ODN protects susceptible BALB/c mice against primary *L. major* infection. Vaccinated and infected mice were able to control a secondary *L. major* challenge since no inflammation and a very low number of parasites were observed in the site of re-infection. Resistance against re-infection correlated with a predominant Th1 response against parasite antigens. Thus, cell cultures established from spleens and the draining lymph node of the secondary site of infection produced high levels of parasite-specific IFN-γ in the absence of IL-4 and IL-10 cytokine production. In addition, re-infected mice showed a high IgG2a/IgG1 ratio for anti-*Leishmania* antibodies. The results suggested that a ribosomal vaccine, which prevents pathology in a primary challenge in combination with parasite persistence, might be effective for long-term maintenance of immunity against CL[78].

*L. major*-derived nuclear protein histone H1 fused with glutathione-S-transferase (GST-H1) and GST antigens were expressed in *E. coli* and purified using GST affinity resin before use in conjunction with montanide ISA 720 as a vaccine against *L. major* challenge in the vervet monkey model of leishmaniasis[79]. Intrademal delivery of an initial dose of 200 µg of the antigen (rGST-H1) mixed with MISA 720 followed by two boosters with the vaccine at a dose of 100 µg of antigen each significantly reduced the development of lesion sizes (providing partial protection) as compared to animal groups that received either GST+MISA 720 or MISA 720 alone at the same dosages. *Leishmania* homologue for receptors of LACK is expressed throughout leishmanial life cycle[80]. Following *L. major* infection, the early LACK-induced IL-4 response appeares to determine disease susceptibility in BALB/c mice, since immunization with *Leishmania* homologue for receptors of LACK promotes the expansion of IL-4 secreting T cells overcoming Th1 responses detrimental to leishmanial parasites[81]. The protective efficacy of *LACK* gene construct was compared with that of LACK protein and IL-12[82]. It was shown that the *LACK* gene construct induced a strong protective response comparable to that achieved when LACK protein plus recombinant IL-12 was administered, and was better than protection seen with LACK protein alone. Moreover, it was demonstrated that the depletion of CD8^+^ cells at the time of vaccination or infection abolished the protective response induced by *LACK DNA* gene construct vaccination, suggesting a role for CD8^+^ T cells in DNA vaccine induced protection toward *L. major*[82]. In susceptible BALB/c mice, immunization with LACK had the ability to control a subsequent infection with *L. major*[83]. To date, the protective efficacy of LACK has been mainly demonstrated in the *L. major* model, but it failed to protect against VL[84]. Hence, researchers sought to manipulate the pathogenic T cell responses to the immunodominant epitope with the use of altered peptide ligands (APLs) and concluded that certain LACK-APLs are able to induce T cell responses with a protective phenotype in an infectious disease such as leishmaniasis[85].

The immunological potential of kinesin protein from the microtubule locus of *L. donovani* was examined as a suitable vaccine candidate. A recombinant protein (rLvacc) was expressed from this region. The antigenicity and immunogenicity studies of this protein by Western blot analysis revealed that rLvacc is strongly recognized by sera from acute VL patients. Following in vitro stimulation of peripheral blood mononuclear cells from cured VL patients with rLvacc and enzyme-linked immunosorbent assay of the supernatant for anti-rLvacc titres, the results showed that immunoglobulin G2 (IgG2) subtype antibodies were predominant, while IgG1 subtype antibodies were produced in very low titers[86]. Vaccination with the DNA construct of this protein generated a good cellular immune response with significant increases in IFN-γ and interleukin-2 (IL-2) cytokine levels (Th1), but no increase in IL-4 levels (Th2). Taken together, the findings suggested that the kinesin motor domain region of *L. donovani* may be considered as a potential vaccine candidate against VL[86]. Proteases have been found to play essential roles in many biological processes, including the pathogenesis of leishmaniasis. The intracellular distribution of a novel *L. donovani* secretory serine protease in the flagellar pocket was determined by immunogold labeling. Flow cytometry and confocal immunofluorescence analysis revealed that the expression of the protease diminishes sequentially from virulent to attenuated strains of this species and is also highly associated with the metacyclic stage of *L. donovani* promastigotes. The level of internalization of parasites treated with the anti-115-kDa-serine protease antibody into host macrophages was significantly reduced from that of non-antibody-treated parasites, suggesting that this serine protease probably plays a role in the infection process. In vivo studies in BALB/c mice confirmed that this 115-kDa serine protease is a potential vaccine candidate delaying infection with *L. donovani*[87]. The researchers referred the mechanism of protection to possible increase in the Th1/Th2 ratio and the involvement of other immunomodulatory cytokines. In a more recent study, *L. donovani* culture derived, soluble, secretory serine protease (pSP) has been shown to be a vaccine target of VL. Protection from VL could be achieved by the use of safer vaccine which generally requires an adjuvant for induction of strong Th1 response. The safety, immunogenicity and efficacy of pSP+IL-12 as a vaccine candidate was assessed in mouse model[87]. BALB/c mice immunized with pSP+IL-12 were protected significantly from challenged infection, even after four months, by reduced parasite load in the liver and spleen and suppressed the development of the disease, along with an increase in IgG2a antibody level in serum, enhanced DTH and strong T-cell proliferation. Groups receiving pSP+IL-12 had augmented pSP antigen-specific Th1 cytokines like IFN-γ and TNF-α response with concomitant decrease of Th2 cytokines, IL-4 and IL-10 after vaccination. In this study, the vaccine efficacy of pSP was further assessed for its prophylactic potential by enumerating matrix metalloprotease-9 (MMP-9) profile, which has been implicated in various diseases. MMP-9 associated with different microbial infections is controlled by their natural inhibitors (TIMPS) and by some cytokines. In this study, pSP was found to regulate excessive inflammation by modulating the balance between MMP-9 and TIMP-1 expression. This modulatory effect has also been demonstrated by IFN-γ mediated downregulation of TNF-α induced MMP-9 expression in activated murine macrophages. This is the first report where a secretory *L. donovani* serine protease (pSP) adjuvanted with IL-12 could also act as protective immunogen by modifying cytokine mediated MMP-9 expression in experimental VL. These findings elucidate the mechanisms for the regulation of MMP-9 following infection of *L. donovani* in vaccinated animals and thus pave the way for developing new immunotherapeutic interventions for VL[88].

The enzyme sterol 24-c-methyltransferase (SMT) is required for the biosynthesis of ergosterol, the major membrane sterol in *Leishmania* parasites. Sterol 24-c-methyltransferase and ergosterol are not found in mammals, so this protein may be an attractive target for anti-leishmanial vaccines and drugs[89]. The researchers demonstrated that SMT from *L. infantum*, which causes VL, is a protective antigen against this parasite[90]. In this study, the vaccine consisted of 10 µg of recombinant SMT (rSMT) plus 20 µg of MPL-SE derivered in C57BL/6 mice. The control mice were given either MPL-SE alone or 10 µg of rSMT. Mice immunized with this vaccine candidate plus MPL-SE showed Ag-specific Th1 immune responses characterized by robust production of IFN-γ upon specific Ag re-exposure in vitro. Because this protein is highly conserved among *Leishmania* species, the researchers evaluated the potential use of SMT to cross-protect against a different form of leishmaniasis. Formulated with MPL-SE, SMT was found to protect mice from CL caused by *L. major*[89]. Mice immunized with the SMT/MPL-SE vaccine developed significantly smaller lesions following ear challenge with *L. major*. The researchers deduced that the vaccine preparation induced antigen-specific multi-functional CD4^+^ T cells capable of producing IFN-γ, IL-2, and/or TNF-α upon antigen re-exposure. They suggested that SMT is a promising vaccine antigen for multiple forms of leishmaniasis. The *Leishmania*-derived recombinant polyprotein, Leish-111f, multicomponent vaccine, has been assessed in clinical trials. Leish-111f is a single polyprotein composed of three molecules fused in tandem: the *L. major* homologue of eukaryotic thiol-specific antioxidant, TSA; the *L. major* stress-inducible protein-1, LmSTI1; and the *L. braziliensis* elongation and initiation factor, LeIF[25]. Initial immunization trials in mice demonstrated that Leish-111f was able to protect mice against *L. major* and *L. amazonensis* infection[91]. There is some evidence that the Leish-111f vaccine can also induce partial protection against VL in animal models; however, it failed to protect dogs against infection and did not prevent disease development in dogs[92]. The Leish-111f or its three component proteins have previously been demonstrated to be efficacious against cutaneous or mucosal leishmaniasis in mice, nonhuman primates, and humans[91]. In another study, the Leish-111f was reported as a vaccine antigen candidate against VL caused by *L. infantum*. The Leish-111f+MPL-SE product reported in this study is the first defined vaccine for leishmaniasis in human clinical trials and has completed phase 1 and 2 safety and immunogenicity testing in normal, healthy human subjects[93]. Analysis of the cellular immune responses of immunized, uninfected mice demonstrated that the vaccine induced a significant increase in CD4^+^ T cells producing IFN-γ, IL 2, and TNF cytokines, indicating a Th1-type immune response. A slightly improved version of the original construct, Leish- 110f, has also been tested in dogs as a therapeutic vaccine in combination with chemotherapy and has led to a reduced number of deaths and higher survival probability[94].

### Naked DNA vaccines

Immunization with naked DNA is a relatively new approach, which promises to revolutionize the prevention and treatment of infectious diseases[95],[96],[97],[98]. In this approach, genes encoding the target proteins are cloned into a mammalian expression vector, and the DNA is directly injected intradermally or intramuscularly. DNA vaccines include single or multiple antigenic DNA molecules using combination of plasmids encoding various antigens. These vaccines are extremely safe as they do not contain any pathogenic organism that may revert in virulence[99]. The mechanism by which DNA vaccination generates potent immune responses appears to be through the activation of innate immune responses by the non−methylated DNA sequences of bacteria and the intense replication within the host, leading to the expression of the recombinant proteins for longer periods. DNA is a very stable molecule, especially when compared to recombinant or live attenuated vaccines. Being stable greatly facilitates storage and distribution in tropical settings with limited health infrastructure. The huge costs associated with the cold chain delivery system of the vaccine from the laboratory to the persons immunized with this vaccine may be an offset. The vaccine need not be kept in a cold or freezing container to maintain its efficacy and, because DNA vaccines are stable, the costs of this delivery system will be negligible. Administration is also easy and multiple plasmids can be combined for the elaboration of multivalent vaccines[64]. There have been several studies conducted on potential DNA vaccines against Leishmania. Handman et al.[100] demonstrated that DNA vaccines can be used therapeutically to treat CL caused by L. major in both genetically resistant C3H/He mice and susceptible BALB/c mice. This is an important finding which demonstrates that DNA vaccines may have a role to therapeutically cure disease in both susceptible and resistant individuals. In previous studies, vaccinations with DNA encoding gp63, LACK, and PSA−2 all protected both genetically resistant and susceptible mice from infection with L. major[74],[82],[101]. Protection was accompanied by Th1 immune responses. Unexpectedly, protection induced by LACK depended on CD8+ T cells, and depletion of this population abrogated protection[82]. In more recent studies, Rodriguez−Cortes et al.[10] found that a multiantigenic DNA vaccine encoding KMII, TRYP, LACK and gp63 did not protect dogs against L. infantum experimental challenge, despite the hypothesis that an effective immune response was more likely to be generated following exposure to more than one antigen. Conversely, Carter et al.[102] found that intramuscular DNA vaccination against the parasite enzyme gammaglutamylcysteine synthetase conferred protection against L. donovani in BALB/c mice. The results of these studies indicate that multiantigenic vaccines do not necessarily confer better protection against Leishmania infection. However, these studies used different species of Leishmania so it is not possible to directly compare the findings.

### Live-attenuated *Leishmania* vaccines

In theory, avirulent microorganism can be generated by defined genetic alteration, eliminating the risk of parasite reversion to the virulent phenotype. By mimicking the natural infection, live-attenuated parasites can deliver a complete spectrum of antigens to the antigen presenting cells, in principle leading to a better immune response that results in a better protective outcome than that observed following immunization with a subunit vaccine[65]. With only a small number of vaccination studies having so far tested *Leishmania* attenuated strains, the live-attenuated anti-leishmanial vaccine is still at its early stages of development. Vaccination with dihydrofolate reductase thymidylate synthase (dhfr-ts) knockout parasites led to protection in a mouse model[103], but failed to protect monkeys against *L. major* infectious challenge[24],[104]. Deletion of serine protease (CP) in *L. mexicana* led to an attenuated strain capable of triggering partial protection against homologous challenge in animal models[105]. These moderately encouraging results were thought to be due to rapid elimination of parasites by the host, since knockout parasites were not persistent. Conversely, *L. major* parasites lacking the *lpg2* gene persisted in mice without pathology and were able to confer protection against homologous infection[106]. However, over time these mutants regained their ability to cause disease in the absence of the *lpg2* gene through an unknown compensatory mechanism[107], suggesting that persistence may not be a desirable feature of a live-attenuated vaccine. Recently, *L. donovani* centrin null mutants (LdCEN-/-) have been reported to have selective growth arrest in the amastigote stage of development, but were viable in culture as promastigotes[108]. Centrin is a calcium-binding cytoskeletal protein involved in the duplication of centrosomes in higher eukaryotes. These mutants were unable to survive in vitro in human macrophages, and animals vaccinated with LdCEN-/- mutants were protected against homologous as well as heterologous challenge. The lack of centrin expression in axenic amastigotes affected specifically their growth in vitro as well as in macrophages. Such a defect in survivability in macrophages may be an indication of the lack of parasite virulence, and therefore these LdCEN-/- parasites could be further tested as a potential vaccine against leishmaniasis[109]. Mice immunized with LdCEN-/- cells showed clearance of virulent challenge parasites in 10 weeks after challenge, with significantly reduced parasite burden in the spleen and no parasites in the liver. In contrast, high parasite loads were observed in the challenged mice previously immunized with heat-killed parasites, or unimmunized. At 10 weeks post virulent challenge, the immunized mice displayed, among the CD4^+^ T-cell population, a significant increase of single and multiple Th1 type cytokine (IFN-γ, IL-2, and TNF-α) producing T cells and increase in IFNγ/IL-10 ratio compared to non-immunized naive mice infected with wild type parasites. The naive challenged mice displayed a reduced Th1 response and increased IL-10, a Th2 polarization accompanied by increased parasite burden in the organs. LdCen-/- immunized mice additionally showed increased IgG2a and NO production[110]. It was demonstrated that *L. major* phosphomannomutase (PMM) deficient mutants were able to protect susceptible mice against infection via an increased magnitude of T cell responses and suppression of IL-10 and IL-13 production early during infection[111]. *L. tarentolae* parasites expressing the *A2* virulence gene have been evaluated as a novel candidate vaccine against murine VL[20]. The results indicated that a single intraperitoneal administration of the A2-recombinant *L. tarentolae* strain protected BALB/c mice against *L. infantum* challenge and that protective immunity was associated with high levels of IFN-γ production prior to and after challenge. More recently, the immunoprotective ability of an *L. infantum* deletion mutant lacking HSP70 type II gene was evaluated as a live vaccine against murine *L. major* disease[112]. Administration of the mutant parasite either by intraperitoneal, intravenous or subcutaneous route in the BALB/c model invariably led to the production of high levels of NO and the development of type 1 immune responses that were protective against *L. major* challenge. The vaccine was also associated with high safety levels. Another approach to attenuating *Leishmania* parasites is the addition of suicide cassettes that lead to the death of the parasite in response to external stimuli[24]. An example of which is the introduction of drug-sensitive genes such as *Saccharomyces cerevisiae* cytosine deaminase gene which is sensitive to 5-fluorocytosine[113]. It has been suggested that parasites carrying drug-sensitive cassettes could provide suitable candidates for leishmanization as an effective treatment of non-resolving lesions could be guaranteed[113]. Alternatively, parasites can be modified to produce biological substances that activate immune attack, such as granulocyte macrophage colony stimulating factor (GM-CSF)[114]. Attenuated vaccines offer a novel approach to immunization against leishmaniasis; however, there are fears that the parasite may revert to a virulent form. Furthermore, targeted deletion of essential virulence genes can result in complete destruction of the parasite or mutants that only delay lesion development[30]. These problems may make the use of killed parasites more attractive for vaccine candidates.

### Development of canine *Leishmania* vaccines

*Leishmania* animal reservoirs pose a big obstacle in the control of human leishmaniasis. Eliminating animal reservoirs has been an essential public health tool for the control of many zoonotic diseases[115],[116]. Canines, particularly domestic dogs, are the main reservoir for VL species and are considered the main source of zoonotic transmission to humans. The development of an effective CVL vaccine represents a cost-effective tool for interrupting the transmission cycle and controlling zoonotic VL infection in humans. CVL is widespread throughout South America[12] and the Mediterranean[13] where *L. infantum* is the most significant causative agent of disease. Asymptomatic infection is common in dogs and, as a large reservoir of parasites are present in the skin, asymptomatic animals are a major source of infection for vector transmission[21]. Human VL is an emerging disease in many areas of the world, including Northern Europe[117] and North America[118], and the spread of VL into nonendemic areas is often preceded by increased incidence of canine infections. There is concern that increased mobility of dogs and changes in vector habitat will result in increased transmission of human VL in previously nonendemic areas[119]. Treatment of CVL shows low efficacy with drugs successfully used for human VL chemotherapy, and drug treatment of dogs rarely results in cure[120]. Control programs for CVL have a demonstrated capacity to reduce the prevalence of human VL disease following interventions that target dog populations in endemic regions[121]. However, these public health campaigns are often complex and expensive to maintain, leading to varying degrees of efficacy. The use of insecticide-impregnated collars can reduce the risk of contracting CVL[122], but is costly and difficult to implement at the national level. The culling of seropositive dogs has long been recommended in Brazil; however, this approach has not led to a reduction in the number of human VL cases and may be of limited value[123]. Therefore, the development of vaccines against CVL is an attractive approach to controlling infection in dogs, reducing the parasite reservoir and thus reducing the risk of transmission of VL to human populations. Immunological characterisation of CVL reveals cellular and humoral immune responses comparable to human infection, including immune dysregulation and increased IL-10 which is associated with disease manifestation and progression[124]. Disease resistance is associated with strong Th1-type immune responses, including IFN-γ expression by antigen-specific T cells. Thus, analogous to a human VL vaccine, an effective CVL vaccine needs to induce strong and long-lasting cell-mediated immunity. Adjuvant choice must be carefully considered for CVL interventions, as live BCG is not appropriate for use in dogs and the identification of appropriate and effective adjuvants will be essential for safe and effective CVL vaccines[125]. In addition, sand fly components are being considered for inclusion in CVL vaccine. Reactive antibodies to two sand fly saliva components (LuLo-D7 and LuLo YELLOW) were identified in infected dogs and proposed as possible vaccine candidates against CVL[126]. Evaluation of a killed *Leishmania* vaccine containing sand fly saliva extract indicated that the vaccine is highly immunogenic and provided support for further development of saliva components as candidates for anti-VL vaccine[127]. This is supported by vaccination studies using the hamster VL model, showing that the salivary protein LJM19 was able to protect hamsters from fatal infection with *L. infantum*[128]. In addition, immunization with salivary proteins LJM17 and LJL143 induced strong cellular and humoral responses in dogs and might be an advantageous addition to anti-CVL vaccine[129]. Currently, there are two commercially available CVL vaccines, *Leishmune* and *Leishtec*. New vaccines under development include recombinant antigen vaccines and both live and killed whole-cell vaccines. The *Leishmune* vaccine produced by Fort Dodge Animal Health was the first commercially licensed vaccine for CVL and has been available in Brazil since 2004[130]. This vaccine consists of the fucose mannose ligand (FML) isolated from *L. donovani* plus a saponin adjuvant. FML is a glycoprotein mixture, and the surface glycoconjugate GP36 is the major immunogen component[57]. This vaccine induced a significant and strong protective effect during phase III trials in dogs living in a VL-endemic area in Brazil with a vaccine efficacy as high as 80%[59],[131]. This protection lasted up to 3.5 years following vaccination, indicating induction of a long-lasting immunity[59]. Since *Leishmune*-vaccinated dogs showed a complete absence of parasites, they are noninfectious and this contributes to the breakdown of the zoonotic VL transmission cycle[132]. During phase III trials of *Leishmune*, there was a concomitant reduction in human VL cases in districts where dogs were vaccinated[131], demonstrating that CVL vaccination interrupts the transmission of disease to humans. FML antigens are present on the surface of *Leishmania* parasites throughout the life cycle, and antibodies raised in vaccinated dogs prevented the binding of procyclic promastigotes to the sand fly midgut[133]. Thus, *Leishmune* acts as a transmission blocking vaccine by clearing parasites from the animal reservoir and preventing the survival of the parasite in the sand fly vector. Currently, the *Leishmune* vaccine is used as a prophylactic and is recommended for asymptomatic noninfected dogs. However, studies show that Leishmune is effective as a therapeutic vaccine for naturally infected dogs[134], particularly when given in combination with chemotherapy[135]. Emerging wide-scale field studies reveal that *Leishmune* decreases the incidence of both human and canine VL after dog vaccination with *Leishmune*[136]. Leish-Tec, another canine vacine is being commercially developed by Hertape Calier Saude Animal and consists of adenovirus expressing the *L. donovani* A2 antigen. Whilst the results from phase-III trials of Leish-Tec are yet to be published, it is known that immunization with a recombinant A2 protein elicits protection against the onset of clinical VL in experimental dog infections[137]. The recombinant adenovirus encoding the A2 gene was capable of inducing strong Th1-type immune responses in vaccinated mice and reduced parasite burdens following challenge with VL parasites[138]. Together, these studies indicate that A2 is an important candidate antigen for the development of CVL vaccines, and future studies should report the impact of this intervention on both canine and human VL infection. As many of the clinical and immunological features of CVL are similar to those observed in human VL, experimental challenge in dogs represents a useful system for evaluating the efficacy of vaccine candidates. The Leish-111f + MPL-SE vaccine is a leading vaccine candidate from human VL and has shown therapeutic efficacy in recent CVL trials[139]. Live attenuated parasite vaccines are also being explored in canine models, including a drug-attenuated line of *L. infantum* established by culturing promastigotes under gentamicin pressure. The attenuated *L. infantum* vaccine strain did not induce clinical symptoms of VL in dogs and provided protection from subsequent challenge with live virulent *L. Infantum*[140]. The elimination of human VL will be difficult to achieve in the presence of persisting animal reservoirs, and veterinary intervention is an important tool for reducing the global burden of human VL disease. The identification of measurable and reliable biomarkers of immunogenicity and protection induced by CVL vaccines may also be informative for human VL vaccine efforts.

## CONCLUSION

Preventive vaccines are recognized as the best and most cost-effective protection measure against pathogens, and are saving millions of lives every year across the globe. *Leishmania* vaccine development has proven to be a difficult and challenging task, which is mostly hampered by inadequate knowledge of parasite pathogenesis and the complexity of immune responses needed for protection. It is highly unlikely that a successful antileishmanial vaccine will be based on a single antigen. Combination vaccines composed of multiple antigens and well-developed adjuvants, such as Leish-111f and MPL-SE and Montanide ISA 720 have the best chances to succeed. Additional clinical trials should soon provide important information on the potential use of this combination. Considering the poor protective efficacy of killed vaccines and difficulties in formulating a subunit vaccine, the use of live-attenuated strains represents a promising alternative. At the moment, major safety concerns and manufacturing considerations place this type of anti-*Leishmania* vaccines in the distant future. Our understanding of T cell determinants needed for long-lasting protective immunity, while still fragmentary, offers hope for development of new strategies for effective T cell vaccines. The main concerns are reliable correlates of immunity that need to be developed in order to evaluate vaccines, and the development of an efficient delivery system and improved adjuvants. Additionally, the elimination of human VL will be difficult to achieve in the presence of persisting animal reservoirs, and veterinary intervention is an important tool for reducing the global burden of human VL disease. The identification of measurable and reliable biomarkers of immunogenicity and protection induced by CVL vaccines may also be informative for human vaccine efforts. Given the rapid progress in the fields of parasite immunology and genetic engineering, a successful anti-*Leishmania* vaccine should be achievable in the near future. Based on the past and present experience on *Leishmania* vaccine studies, it appears that future experiments should include appropriate adjuvants as components in order to achieve effective vaccines against human leishmaniasis.
